# A Case Report of Isolated Bilateral Cerebral Peduncular Infarction

**DOI:** 10.1155/2017/9845917

**Published:** 2017-07-31

**Authors:** Chenguang Zhou, Yuanhong He, Xiaorui Tian, Zhiwen Chao, Yinghui Zhu, Du Cheng, Kui Li

**Affiliations:** Department of Neurology, The Fifth Affiliated Hospital of Zhengzhou University, Zhengzhou, Henan 450052, China

## Abstract

Isolated bilateral cerebral peduncular infarctions (BCPI) presenting as acute pseudobulbar palsy are rarely reported and, to the best of our knowledge, most of the previous reports of BCPI were related to locked-in syndrome and disturbance of consciousness. Herein, we described a case of a 55-year-old man who presented with acute pseudobulbar palsy and mild tetraparesis, but preserved eye movements, with no consciousness disturbance. DWI revealed an acute infarction involving the central portion of the cerebral peduncle with a characteristic “traditional Chinese eight character” sign. The relationship between the infarcted range in the cerebral peduncle and the clinical manifestation was discussed in our report.

## 1. Introduction

Bilateral cerebral peduncular infarctions (BCPI) are an extremely rare neurological disorder, occurring in only 0.26% of patients with acute ischemic stroke [1]. The anatomic etiology is presumed to be the occlusion or stenosis of the large vertebrobasilar artery. Several cases of BCPI have been reported earlier to be associated with locked-in syndrome and disturbance of consciousness [2–6]. However, cerebral peduncle lesions in such cases were accompanied with infarctions in other regions such as pons, cerebellum, and other posterior cerebral artery (PCA) territories. To the best of our knowledge, isolated bilateral BCPI demonstrated by MRI is rare and has hitherto been described in only 1 patient before this article [6]. Herein, we reported a patient presenting with acute pseudobulbar palsy, mild tetraparesis, and intact eye movements, which resulted from isolated and symmetric BCPI mainly involving the central portion of the cerebral peduncle.

## 2. Case Report

**Figure 1:**
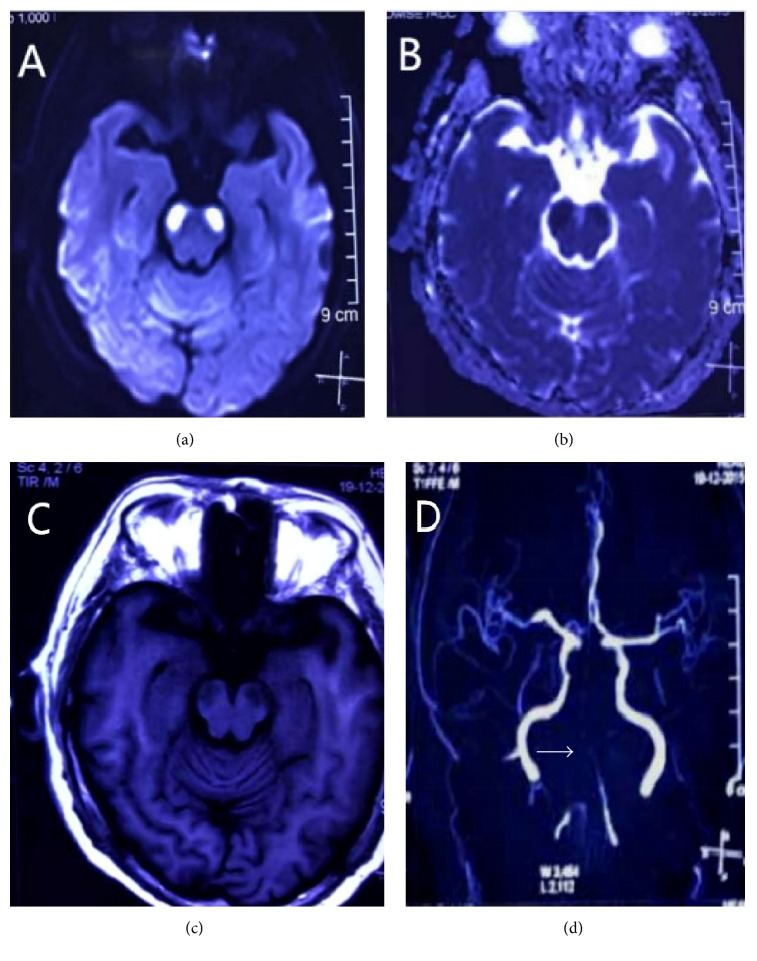
(a) Diffusion-weighted image showing high signal intensity in bilateral cerebral peduncles, with corresponding hypointensity on apparent diffusion coefficient map (b). T1-weighted (c) magnetic resonance imaging showing low intensity in bilateral cerebral peduncles with sparing of the medial and most of the lateral portion with low signal intensity. The entire basilar and distal vertebral arteries (white arrow) are not shown on the magnetic resonance angiography (MRA) (d).

A 55-year-old man with known longstanding hypertension and diabetes mellitus type 2 presented with acute onset of dizziness, slurred speech, and unsteady gait. On admission, his blood pressure was 178/100 mmHg and heart rate was 92 beats/min. He was dispirited but oriented. Additionally, he did not take the initiative to speak but could answer simple questions in a whisper with a few words. Neurological examination revealed severe dysphagia, dysarthria, mild tetraplegia (Medical Research Council Grade 4-5), and ataxia of the trunk and all four limbs. Pupillary reflexes and extraocular movements (both vertical and horizontal) were intact. Bilateral Babinski signs were positive. Meanwhile, he also showed spasmodic crying and laughing on examination. Magnetic resonance imaging (MRI) of the brain performed 36 hours after the onset of symptoms revealed acute, bilateral, symmetric, cerebral peduncular infarcts (BCPI), which showed increased signal intensity on diffusion-weighted imaging (DWI) with low values on apparent diffusion coefficient (ADC) map (Figures 1(a) and 1(b)), in line with acute infarction. T1-weighted images (T1WI) demonstrated the lesion mainly involving the central portion of the cerebral peduncle with low signal intensity (Figure 1(c)). Magnetic resonance angiography (MRA) disclosed the vertebrobasilar artery occlusion and the absence of flow signals of bilateral PCA without collateral patency of the posterior communicating artery (Figure 1(d)). The patient was given antiplatelet and hypervolemic therapy, with no further deterioration of his condition. He was transferred to the rehabilitation ward for rehabilitation treatment of swallowing function twenty days after his admission.

## 3. Discussion

**Figure 2:**
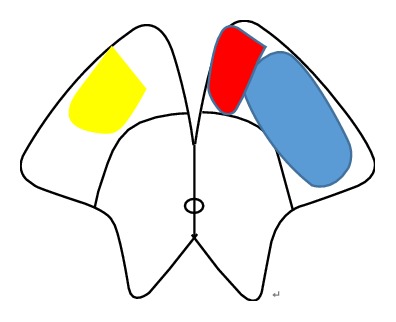
Schematic diagram of the involved areas of cerebral peduncular infarction showing the present case on the right side (yellow), the case from Dehaene and Dom's report (red + blue), and the case from Tarek Zakaria's report (blue) on the left side.

Previously, a case of bilateral cerebral peduncular infarction involving most of the lateral portion of the peduncle with a sign termed “Mickey Mouse ears” on DWI has been reported [5]. Interestingly, our patient had lesions mainly involving the central portion of the cerebral peduncle, which simulated a “traditional Chinese eight character” sign on DWI. Several cases of BCPI infarctions have been reported previously associated with locked-in syndrome. The term “locked-in syndrome” (LIS) was defined as complete paralysis of bulbar muscles and all extremities with preserved consciousness, leaving the patients with only vertical eye movements and blinking. Pathologically, ventral pontine is the most frequent cause, but LIS due to isolated midbrain lesion is extremely rare. Dehaene and Dom described a patient with LIS including impaired horizontal eye movements which corresponded exactly to the original description of LIS. The autopsy revealed the lesion involving the medial two-thirds of each cerebral peduncle [7] (Figure 2). However, the mesencephalic LIS is not always synonymous with the complete LIS in ventral pontine. A few of mesencephalic LIS cases confirmed by MRI showed intact eye movements with the lesion involving the central and lateral portion of the peduncle [2–4, 6] (Figure 2). Additionally, cerebral peduncle lesions in such cases were accompanied with infarctions in other regions such as pons, cerebellum, and other posterior cerebral artery (PCA) territories. Our present patient showed full eye movement, severe pseudobulbar palsy, and mild tetraplegia, probably because the area of infarction was limited within almost the central portion of the peduncle with the sparing of cortical efferent pathways mediating the voluntary conjugate movements and major corticospinal tracts. Although the exact localization of the cortical efferent pathways mediating the voluntary conjugate movements was unclear, the medial portion of the peduncle was probably related to the voluntary eye movement. Accordingly, we know that the clinical manifestation of BCPI is related to the infarct range of the cerebral peduncle involved.

The circulation of the cerebral peduncle is mainly supplied by perforating branches from the collicular artery, posteromedial choroidal artery, and circumflex branches from the p1 or p2 segment of PCA. In addition, it also receives contribution from the posterior communicating artery and anterior choroidal artery [8]. Despite being rare, Chen et al. reported a study of 14 cases of BCPI, where 12 (85.7%) patients had vertebrobasilar artery severe stenosis or occlusion. 11 patients were diagnosed with large artery atherosclerosis (LAA), 1 was diagnosed with artery-to-artery embolism, and the other 2 were diagnosed with cardiac embolism. The main mechanism of BCPI caused by LAA was hypoperfusion in their study [1]. In our patient, both basilar artery and the bilateral PCA were not shown on the MRA due to the severe decrease in the flow signals. Although there was no documented drop in blood pressure, we speculated that the main mechanism of isolated BCPI in our patient may be related to hypoperfusion rather than thromboembolism, as a result of severe large artery occlusion and lack of evidence of embolus. With the sparing of the cerebellum and occipital lobe in posterior circulation stroke, we hypothesized that the hemodynamics of the tiny terminal branches feeding the cerebral peduncle are more easily affected than large branches providing the irrigation to occipital lobe and other regions in the context of vertebrobasilar artery stenosis and occlusion.

In summary, we reported a case of a patient who presented with acute pseudobulbar palsy, preserved eye movements, and only mild tetraparesis resulting from isolated bilateral cerebral peduncular infarction. It was different from previous reports in which BCPI were associated with locked-in syndrome and disturbance of consciousness. In addition, the hypoperfusion caused by vertebrobasilar artery stenosis probably was the key etiology of BCPI in our study, which was in accordance with the literature.
